# Factors associated with serum CA19-9 levels among healthy children: a cross-sectional study

**DOI:** 10.1186/1472-6890-12-23

**Published:** 2012-12-04

**Authors:** Sayo Kawai, Sueli M Oba-Shinjo, Lucy S Ito, Miyuki Uno, Suely K N Marie, Nobuyuki Hamajima

**Affiliations:** 1 Department of Preventive Medicine, Nagoya University Graduate School of Medicine, 65 Tsurumai-cho, Showa-ku, Nagoya, Japan; 2 Laboratory of Molecular and Cellular Biology, Department of Neurology, School of Medicine, University of São Paulo, São Paulo, Brazil; 3 Japanese Brazilian Health Professional Volunteer Group, São Paulo, Brazil

**Keywords:** CA19-9, Healthy children, BMI, *Lewis* and *secretor* gene polymorphisms

## Abstract

**Background:**

CA19-9 is a tumor marker mainly used for biliary tract, pancreas and colorectum. Since the marker applies usually for adults, the normal range of serum CA19-9 among children has been rarely reported. This is the first study reporting the distribution of serum CA19-9 levels among cancer-free children as well as their parents, taking into account the *Lewis* and *secretor* gene polymorphism and physical growth.

**Methods:**

Study subjects were 972 apparently healthy Japanese Brazilians including 476 children aged from 1 to 19 years.

**Results:**

The comparisons in five-year age groups demonstrated that the mean values of serum CA19-9 was lower in the boys than in the girls, and higher in younger age groups; 22.5 U/ml for 1–4 year-old (n=13), 17.4 U/ml for 5–9 year-old (n=36), 15.5 U/ml for 10–14 year-old (n=96) and 10.2 U/ml for 15–19 year-old (n=74) in boys, and 25.3 U/ml (n=11), 27.1 U/ml (n=50), 17.7 U/ml (n=105) and 13.5 U/ml (n=59) in girls, respectively. The difference in those geometric means was statistically significant among four age groups (*p*=0.006, ANOVA adjusted for sex). After *Lewis* and *secretor* genotypes, which are definitive factors of serum CA19-9, were taken into account, geometric mean of serum CA19-9 was associated with any of BMI (*p*<0.001), height (*p*<0.001) and weight (*p*<0.001) among children excluding those with *le*/*le* genotype. The associations were still significant when age was adjusted.

**Conclusions:**

Serum CA19-9 values were higher among children than among adults, and influenced by sex, height, weight, and BMI even after the adjustment for age as well as *Le* and *Se* genotypes.

## Background

CA19-9 is a blood group antigen-related marker for cancer screening and a prognostic factor during treatment [1]. Currently, serum CA19-9 is regarded as the most sensitive and specific marker for the diagnosis and follow-up of pancreatic cancer. The sensitivity of the test to detect pancreatic cancer was 73.3% with 37 U/ml as the cut-off value, the specificity was 87.0%, and the diagnostic accuracy was 60.3% [2]. CA19-9 is also raised in biliary tract [3], colorectum [1,4] and ovarian [5] malignancies. Increased levels of serum CA19-9 can also occur in hydronephrosis [6], and Hashimoto’s Thyroiditis [7].

Since the assay for CA19-9 is using the antibody (1116-NS-19-9) that recognizes the carbohydrate structure sialylated Lewis a (sialyl-Le^a^) structure [8], Lewis phenotype influences the CA19-9 concentration in serum. Two independent genes, *Lewis* gene (*Le* allele or *le* allele) and *secretor* gene (*Se* allele or *se* allele), determine the Lewis phenotype [9]. In those with no enzyme activity genotype (*le*/*le*) of *Lewis* (*Le*) gene, serum CA19-9 values are negative (<1.0 U/ml), while no enzyme activity genotype (*se*/*se*) of *secretor* (*Se*) gene is considered to have an association with high serum CA19-9 levels. In Japanese, the distinction of *le* from *Le* was practically possible for genotyping T59G polymorphism (*59T* for *Le* and *59G* for *le*). For *Se* gene, A385T (*sej*) and pseudogene (*se5*) were used for the detection of *se* alleles [10]. Our previous study showed the geometric mean of serum CA19-9 was significantly associated with current smoking among adults, independently of *Le* and *Se* genotypes [11]. However, since the tumor marker usually applies for adults, the above-mentioned studies were all performed with adult subjects, and there were few reports about the distribution of serum CA19-9 values of children.

To our knowledge, there were only three case–control studies on the distribution, so far. The first study was aimed to establish the reference values of tumor makers including CA19-9 for pediatric malignant diseases. They reported the distribution of serum CA19-9 concentration in 52 healthy children collected for a nutritional study aged 1 to 17 years [12]. Another study reported the mean values of serum CA19-9 among 30 pediatric outpatients without blood diseases as a control group compared with a 5-year-old patient of malignant lymphoma [13]. The other study reported that the mean values of serum CA19-9 among 27 healthy boys and girls whose mean age was 30.1 months, as a control group for 27 patients with unilateral ureteropelvic junction obstruction aged 0.5 to 98 months [14].

In order to make an accurate diagnosis or prognosis of disorders using serum CA19-9 for pediatric patients, it is important to know about the normal values of CA19-9 among children. In this study, we measured the serum CA19-9 values of children and adolescents aged less than 20 years among cancer-free Japanese-Brazilians, as well as those of their parents. The association between serum CA19-9 and candidate factors such as age, sex, weight, height, and body mass index (BMI, weight in kilogram divided by height squared in meter) were also examined.

## Methods

### Study subjects

The subjects of this study were apparently healthy family volunteers, who were enrolled for a study on *Helicobacter pylori* infection through Japanese-Brazilian societies in São Paulo in 2002 [15]. The family units were defined as husband, wife, and one or more children aged from 1 to 19 years and they all lived in the same household. Written informed consent was obtained from all the participants. In case of children, the parents signed the consent form instead of their children providing consent. The collection of lifestyle data and blood was conducted after the consent was obtained. Among the participated 1,037 Japanese Brazilians, 1,024 samples were available for CA19-9 measurement. Excluding the subjects lacking of one or more items of data under study, and one subject with more than 250 U/ml of serum CA19-9 who was on suspicion of carrying some diseases or disorders, 972 subjects (496 adults and 476 children) were applied to the present study. This study was approved by Ethics Committees of Nagoya University School of Medicine (approval number 1112).

### Quantitative analysis of serum CA19-9 and genotyping

A 10-ml sample of venous blood was obtained from each participant. The blood samples were centrifuged and frozen at -20°C according to an identical protocol, then they were transferred to Japan in 2002 and stored at -80°C thereafter. The serum concentration of CA19-9 was measured by SRL Inc., Tokyo Japan, in 2009 with chemiluminescent enzyme immunoassay (CLEIA) (upper limit of normal range, 37 U/ml). DNAs were extracted from the blood by a salting-out method and utilized for polymorphism analysis. The *Le* and *Se* gene polymorphisms were genotyped in 2009 by polymerase chain reaction with confronting two-pair primers (PCR-CTPP) [16]. The detailed primer sets and PCR conditions were shown in our previous study [17].

### Statistical analysis

Information about age, sex, smoking and alcohol drinking habit was self-reported and BMI was calculated from height and weight. Hardy-Weinberg equilibrium was tested for the *Le* and *Se* genotypes. Since the distribution of serum CA19-9 values followed lognormal, geometric means were tested by analysis of variance (ANOVA). When geometric means were calculated, the detectable limit (1 U/ml) was used for the below limit. Box and whisker plot in figures indicates lower adjacent value (the lowest datum still within 1.5 interquartile range of the lower quartile), 25th percentile (lower quartile), 50th percentile (median), 75th percentile (upper quartile), and upper adjacent value (the highest datum still within 1.5 interquartile range of upper quartile). All calculations were performed with the Stata 9 computer program (StataCorp, College Station, TX, USA).

## Results

### Background characteristics and genotype frequencies

Table 1 shows the characteristics of the subjects according to sex. The data indicated that the mean value of serum CA19-9 was higher in females than males. There were no smokers or alcohol drinkers among the 476 children aged less than 20 years. Among their parents, current smokers were 22.6% in males and 9.4% in females, and alcohol drinkers were 62.7% and 25.4%, respectively.

**Table 1:** Characteristics of study subjects

**Characteristics**	**Children aged less than 20 years**		**Adults (Parents)**	
	**Boys**	**Girls**	**Men**	**Women**
	**(N=251)**	**(N=225)**	**(N=252)**	**(N=244)**
Age in years				
Mean (Range)	11.5 (1–19)	11.7 (1–19)	45.8 (25–66)	42.8 (22–69)
CA19-9				
Mean* (±S.D.)	14.8 (±19.4)	19.1 (±21.4)	9.5 (±9.3)	11.9 (±11.9)
Range	1-136	1-128	1–89.8	1–87.2
Height (cm)				
Mean (Range)	147.7 (72–185)	143.8 (60–172)	167.9 (150–190)	156.0 (140–173)
Weight (kg)				
Mean (Range)	44.6 (10–100)	39.6 (10–83)	70.6 (35–105)	56.2 (38–90)
BMI (kg/m^2^)				
Mean (Range)	19.4 (6.1-35.0)	18.6 (5.9-33.3)	25.0 (15.6-36.3)	23.1 (16.9-37.8)
Smoking				
Current smokers	0	0	57 (22.6%)	23 (9.4%)
Non-current smokers	0	0	195 (77.4%)	221 (90.6%)
Alcohol				
Current drinkers	0	0	158 (62.7%)	62 (25.4%)
Non-current drinkers	0	0	94 (37.3%)	182 (74.6%)

NOTE. BMI: body mass index. ^*^Arithmetic mean.

Allele frequency of *Le* T59G polymorphism was 69.4% for *Le* (59T) and 30.6% for *le* (59G), with 475 (48.9%) subjects for *Le*/*Le*, 400 (41.1%) for *Le*/*le* and 97 (10.0%) for *le*/*le*. On *Se* A385T polymorphism and pseudogene, allele frequencies were 52.1% for *A* (*Se*), 42.2% for *T* (*sej*) and 5.7% for pseudogene (*se5*), with 259 (26.7%) for *Se*/*Se*, 495 (50.9%) for *Se*/*se* and 218 (22.4%) for *se*/*se*, where *sej* and *se5* were categorized into one group as “*se*”. There was only one subject with homozygous for *se5* allele (*se5*/*se5*) in this dataset. The genotype frequencies for *Le* and *Se* were in Hardy-Weinberg equilibrium (χ^2^=0.89, *p*=0.34 and χ^2^=0.40, *p*=0.53, respectively). Table 2 shows *Le* and *Se* genotype frequencies of the children and their parents.

**Table 2:** **Genotype frequencies for *Lewis *and *secretor *polymorphisms**

**Characteristics**	**Children**	**Parents**	**All subjects**
*Lewis* T59G			
*Le*/*Le* (*TT*)	229 (48.1%)	246 (49.6%)	475 (48.9%)
*Le*/*le* (*TG*)	199 (41.8%)	201 (40.5%)	400 (41.1%)
*Le*/*le* (*GG*)	48 (10.1%)	49 (9.9%)	97 (10.0%)
*secretor* A385T & pseudogene^*^			
*Se*/*Se*	121 (25.4%)	138 (27.8%)	259 (26.7%)
*Se*/*se*	251 (52.7%)	244 (49.2%)	495 (50.9%)
*se*/*se*	104 (21.9%)	114 (23.0%)	218 (22.4%)

^*^sej (*385T*) and *se5* (pseudogene) are categorized into one group as “*se*”.

### Serum CA19-9 and ages of subjects

**Figure 1:**
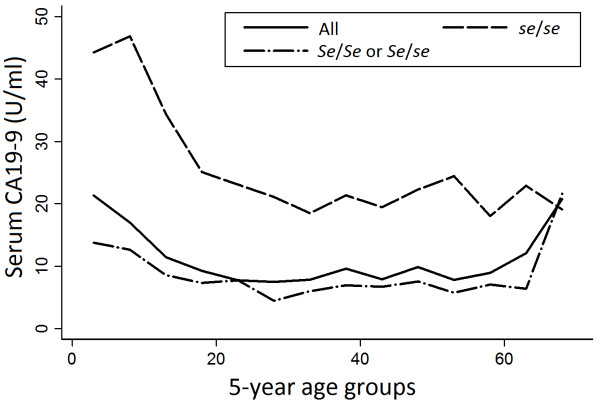
**Geometric mean of serum CA19-9 for 5-year age groups according to *Se *genotype. **Shift of the geometric mean of serum CA19-9 for each 5-year age groups according to *Se* genotype excluding those with *le*/*le* genotype.

The distribution of serum CA19-9 by age is shown in Figure 1. The comparison in five-year age groups demonstrated that the geometric mean of serum CA19-9 was higher in younger age groups; 22.5 U/ml for 1–4 year-old (n=13), 17.4 U/ml for 5–9 year-old (n=36), 15.5 U/ml for 10–14 year-old (n=96) and 10.2 U/ml for 15–19 year-old (n=74) in boys, and 25.3 U/ml (n=11), 27.1 U/ml (n=50), 17.7 U/ml (n=105) and 13.5 U/ml (n=59) in girls, respectively. The difference in those geometric means was statistically significant among four age groups (*p*=0.006, ANOVA adjusted for sex). Regression analysis also indicated that age was significantly associated with logarithm of serum CA19-9 levels (β,−0.022, *p*<0.001). This tendency was not observed among the parents (*p*=0.38, ANOVA adjusted for sex). The significant association between serum CA19-9 and sex was also observed only among children (*p*=0.004 among the children and *p*=0.29 among the parents, ANOVA adjusted for four age groups).

### Serum CA19-9 according to genotypes and generations

**Figure 2:**
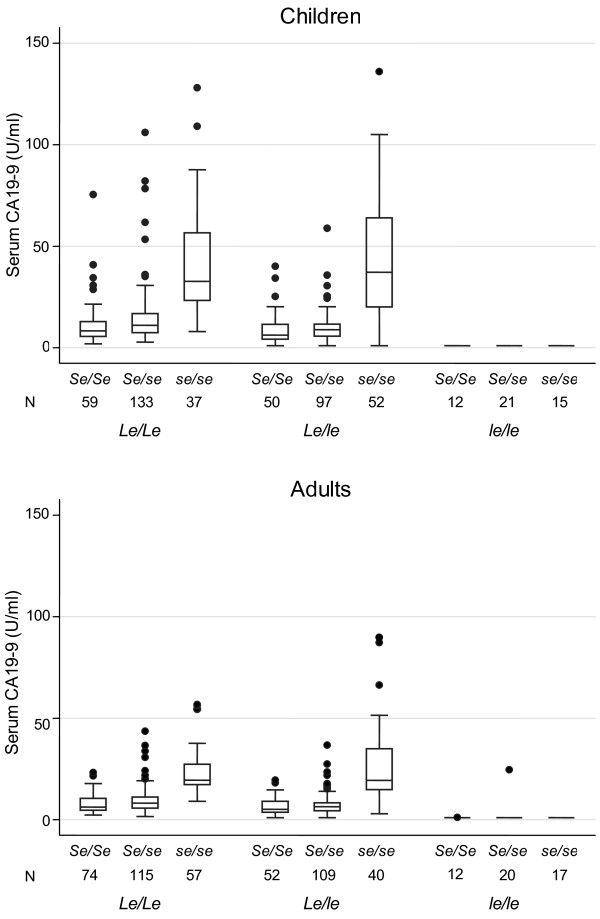
**Distribution of serum CA19-9 according to *Le *and *Se *genotypes.**

Among the children aged less than 20 years, geometric mean for CA19-9 was 34.1 U/ml for *Le*/*Le* &*se*/*se*, 32.6 U/ml for *Le*/*le* &*se*/*se*, 11.6 U/ml for *Le*/*Le* &*Se*/*se*, 8.7 U/ml for *Le*/*Le* &*Se*/*Se*, 8.3 U/ml for *Le*/*le* &*Se*/*se*, 6.4 U/ml for *Le*/*le* &*Se*/*Se*, and below the detection limit (1 U/ml) for *le*/*le* &*se*/*se*, *le*/*le* &*Se*/*se* and *le*/*le* &*Se*/*Se*. Among their parents, the mean for CA19-9 values was 20.9 U/ml, 21.0 U/ml, 8.3 U/ml, 6.8 U/ml, 6.3 U/ml, 5.5 U/ml, below the detection limit, 1.2 U/ml and 1.0 U/ml, respectively. The difference of the geometric mean values between the children and the parents was statistically significant (*p*<0.0001, ANOVA adjusted for sex) (Table 3 and Figure 2).

**Table 3:** Geometric means of serum CA19-9 (U/ml) for each genotype among the children and their parents

**Genotype**	**Children (N)**	**Parents (N)**	***p*-value**^**b**^
*Le*/*Le* &*se*/*se*	34.1 (37)	20.9 (57)	< 0.0001
*Le*/*le* &*se*/*se*	32.6 (52)	21.0 (40)	0.005
*Le*/*Le* &*Se*/*se*	11.6 (133)	8.3 (115)	< 0.0001
*Le*/*Le* &*Se*/*Se*	8.7 (59)	6.8 (74)	0.03
*Le*/*le* &*Se*/*se*	8.3 (97)	6.3 (109)	0.001
*Le*/*le* &*Se*/*Se*	6.4 (50)	5.5 (52)	0.36
*le*/*le* &*se*/*se*	1^a^ (15)	1^a^ (17)	n/a
*le*/*le* &*Se*/*se*	1^a^ (21)	1.2 (20)	0.35
*le*/*le* &*Se*/*Se*	1^a^ (12)	1.0 (12)	0.29
Total	9.3 (476)	7.1 (496)	< 0.0001

^a^All subjects in the groups indicated below the detection limit (1 U/ml)

^b^Calculated by ANOVA adjusted for sex.

### Effects of height, weight, and BMI on serum CA19-9 among the children

Among those with *Le*/*Le* or *Le*/*le* (the subjects excluding those with *le*/*le*), it was found that children aged less than 20 years with high BMI (20 kg/m^2^ and more) indicated lower values of serum CA19-9 (*p*=0.007, ANOVA adjusted for age groups, sex and *Se* genotype) (Table 4). Among the same subjects, the regression analysis indicated that logarithm of CA19-9 was significantly associated with any of BMI (β, −0.018, *p*<0.001), height (β, −0.005, *p*<0.001) and weight (β, −0.006, *p*<0.001) adjusted for sex and *Se* genotype, while the associations were not observed among their parents (*p*=0.31 for BMI, *p*=0.17 for height, and *p*=0.71 for weight). The associations among the children were still significant when they further adjusted for age (*p*=0.022 for BMI, *p*=0.013 for height, and *p*=0.002 for weight) (Table 5).

**Table 4:** **Geometric means of serum CA19-9 for body mass index (BMI) among the children excluding those with *le*/*le *genotype**

	**N**	**Geometric mean (U/ml)**	F^*^	***p*-value**
BMI (kg/m^2^) < 20	275	13.6		
BMI (kg/m^2^) ≥ 20	153	9.6	7.31	0.007

^*^Calculated by ANOVA adjusted for age group, sex and *Se* genotype.

**Table 5:** **Coefficients (β) of BMI, height and weight, estimated by regression analysis for the logarithm of CA19-9 among the subjects excluding those with *le*/*le *genotype**

	**β (*p*-value)**	**β (*p*-value)**
	**adjusted for sex &*Se *genotype**	**adjusted for sex, *Se *genotype & age**^**a**^
Children		
BMI	−0.018 (<0.001)	−0.010 (0.022)
Height	−0.005 (<0.001)	−0.003 (0.013)
Weight	−0.006 (<0.001)	−0.005 (0.002)
Parents^b^		
BMI	0.004 (0.31)	0.003 (0.39)
Height	−0.003 (0.17)	−0.002 (0.25)
Weight	0.001 (0.71)	0.001 (0.70)

^a^Five-year age groups.

^b^Drinking and smoking statuses are added as adjusting factors

## Discussion

There were few reports on serum CA19-9 values of children. Except for the case–control studies with a limited number of controls, this is the first report of the distribution of serum CA19-9 values of subjects aged less than 20 years. The new finding is that the children in early years indicated high serum CA19-9 values and it decreases along with their maturation.

Since *Le* and *Se* genotypes influence serum CA19-9 levels, the genotypes were taken into account for the present study. The allele frequencies were similar to those of the previous studies for Japanese population [10,18]. In *Le* gene, *le/le* genotype has no enzyme activity, which results in no synthesis of CA19-9 even among cancer patients. In *Se* gene, 385T (*sej*) and pseudogene (*se5*) are no activity alleles, so that *sej*/*sej*, *sej*/*se5*, and *se5*/*se5* genotypes are concerned with over synthesis of CA19-9. This study reported *Le* and *Se* genotype frequencies and the geometric mean values of serum CA19-9 for each combination of these genotypes. The combination of the full enzyme activity genotype of *Le* gene (*Le*/*Le* or *Le*/*le*) and no enzyme activity genotype of *Se* gene (*se*/*se*) indicated the highest mean value of serum CA19-9 among both of the generations.

In case of Caucasians, although the reported polymorphisms of *Le* and *Se* genes are partly different from Japanese, the alleles without enzyme activity are observed. The common haplotypes without enzyme activity are *59G* &*508A* (*le1*) and *59G* &*1067A* (*le2*) in Japanese, and *59G* &*314T* in Caucasians [19]. Any of *508A*, *1067A* and *314T* alleles of *Le* gene could lead to lose the Lewis enzyme activity. Concerning *Se* gene, common alleles without enzyme activity are *sej* and *se5* among Japanese, and *428A* of G428A polymorphism in Caucasians. Although there was no previous information about the frequencies of *Le*, *Se* genotypes among Japanese-Brazilians, their genetic background is basically the same and this study showed the similar frequencies to the previous Japanese population. Since there are no substantial differences in frequency of no activity alleles between Caucasians and Japanese, the consequence of this study would be applicable to any population groups.

The present study showed that serum CA19-9 levels were significantly higher in children than in their parents, and it seemed to be associated with body constitutions or maturity. The mean serum CA19-9 value of the parents of children whose CA19-9 values were higher than 37 U/ml was 12.3 U/ml and those of the rest of parents was 10.4 U/ml; the difference was not statistically significant (*p*=0.19, ANOVA adjusted for age groups, sex and *Le* and *Se* genotypes). In this study, the youngest group indicated the highest mean value of serum CA19-9 in five-year age groups of the children. The regression analysis showed that younger age and high serum CA19-9 level were significantly associated among the children (*p*<0.001). This result was inconsistent with the study in Finland about 20 years ago, which was concluded that the age of individuals did not have any influence on the concentrations of the CA19-9 marker [12]. However, they also reported that the range of serum CA19-9 levels in healthy children were clearly wider than in adults and there were no detailed information about the mean values of each age groups.

At the beginning, we thought the feasible candidate for explaining the association between younger age and high serum CA19-9 was BMI. ANOVA demonstrated that children with high BMI (20 kg/m^2^ and more) indicated significantly lower values of serum CA19-9 (*p*=0.007). Then we conducted the regression analysis with BMI, as well as height and weight, to test the hypothesis that the tendency could be associated with body constitutions. Any association with BMI, height and weight was significant, and the associations were still significant when they adjusted for age. Therefore, BMI seems to be one of the factors to determine the serum CA19-9 levels independently of ageing. Recently, one study reported that obese men had lower tumor marker levels, including CA19-9, than men in normal weight. They considered that hemodilution from increased plasma volume was the main reason of the observed decrease in tumor marker concentration in men with a high BMI [20]. However, decreasing of serum CA19-9 was not the results for obese among the present study subjects, there could be other unknown factors we should focus on.

## Conclusions

Serum CA19-9 values were higher among children than among adults, and influenced by sex, height, weight, and BMI even after the adjustment for age as well as *Le* and *Se* genotypes. Further investigation would be required to elucidate the mechanism of this phenomenon.

## Abbreviations

BMI: Body mass index; CIEIA: Competitive inhibition enzyme immunoassay; PCR-CTPP: Polymerase chain reaction with confronting two-pair primers; *Le* gene: *Lewis* gene; *Se* gene: *Secretor* gene; ANOVA: Analysis of variances.

## Competing interests

The authors have no conflicts of interest and did not receive any outside assistance writing this manuscript.

## Authors’ contributions

SK performed the statistical analysis and drafted the manuscript. SMO, LSI, MU, and SKNM have made substantial contributions to study design, acquisition of data, and manuscript preparation. NH conceived of the study. All authors read and approved the final manuscript.

## Pre-publication history

The pre-publication history for this paper can be accessed here:

http://www.biomedcentral.com/1472-6890/12/23/prepub
